# Hyperspectral Image Change Detection Method Based on the Balanced Metric

**DOI:** 10.3390/s25041158

**Published:** 2025-02-13

**Authors:** Xintao Liang, Xinling Li, Qingyan Wang, Jiadong Qian, Yujing Wang

**Affiliations:** School of Measurement-Control and Communication Engineering, Harbin University of Science and Technology, Harbin 150080, China; lxt3@163.com (X.L.); 2220610158@stu.hrbust.edu.cn (X.L.); qicaitunono@gmail.com (J.Q.); mirrorwyj@hrbust.edu.cn (Y.W.)

**Keywords:** hyperspectral image, change detection, Siamese network, metrics learning

## Abstract

Change detection, as a popular research direction for dynamic monitoring of land cover change, usually uses hyperspectral remote-sensing images as data sources. Hyperspectral images have rich spatial–spectral information, but traditional change detection methods have limited ability to express the features of hyperspectral images, and it is difficult to identify the complex detailed features, semantic features, and spatial–temporal correlation features in two-phase hyperspectral images. Effectively using the abundant spatial and spectral information in hyperspectral images to complete change detection is a challenging task. This paper proposes a hyperspectral image change detection method based on the balanced metric, which uses the spatiotemporal attention module to translate bi-temporal hyperspectral images to the same eigenspace, uses the deep Siamese network structure to extract deep semantic features and shallow spatial features, and measures sample features according to the Euclidean distance. In the training phase, the model is optimized by minimizing the loss of distance maps and label maps. In the testing phase, the prediction map is generated by simple thresholding of distance maps. Experiments show that on the four datasets, the proposed method can achieve a good change detection effect.

## 1. Introduction

Satellite remote-sensing technology in recent years can be characterized as macro, comprehensive, rapid, dynamic, and accurate. It is commonly used to investigate Earth resources, observe regional environmental changes, and study the change in the global environment [1]. Remote sensing is the most advanced means to acquire land cover information, and it has many merits, such as its real-time, objective, and wide coverage [2]. Since the 1990s, remote-sensing image technology has performed well in various applications, such as urban development planning, natural resource investigation and monitoring, and fine agricultural identification [3,4,5].

Utilizing hyperspectral images can allow for detecting substances with diagnostic spectral absorption characteristics and simultaneously allow for imaging the target area through fine and continuous spectral bands. There is presumably a continuous spectrum of surface objects in the imaging area [6], and hyperspectral image data can be vividly described by “three-dimensional data blocks”. In the two-dimensional plane coordinate system, the wavelength information axis of each pixel is added, which integrates image spatial and spectral information. This has high spectral and spatial resolutions, thus providing abundant ground object information [7]. Given their wide coverage and rich spatial and spectral information, hyperspectral images are becoming the main means of ground observation, and using multi-temporal hyperspectral data for dynamic monitoring of land cover changes is a popular research direction in remote sensing [8].

Hyperspectral change detection is based on bi-temporal hyperspectral image data collected from two different periods and covering the same position, and it involves analyzing the dynamic changes in the ground surface. Specifically, labels are assigned to each pixel in an image, which indicates whether the category of ground objects of the corresponding pixel changes [9]. The traditional hyperspectral image change detection method focuses on the generation of difference maps. Two images are directly subjected to algebraic operations, to obtain a difference map and final change detection result [10]. The image preprocessing before this step has a significant impact on the detection accuracy. Hyperspectral images have a high spectral resolution and contain a large amount of spectral information, which inevitably causes a lot of information redundancy in change detection applications. In order to ensure both a high accuracy and high efficiency in change detection of hyperspectral images, Ref. [11] uses principal component analysis to map hyperspectral data to the feature space in order to label change regions. Ref. [12] proposed a multi-scale morphological compression change vector analysis method, which uses morphological computation to process spatial neighborhood information and eliminate noise. In response to the slow change in semantic invariant pixel spectral features in multi-temporal images, Ref. [13] constructed three slow feature analysis methods to extract the feature components with the strongest temporal invariance for change analysis. But it destroys the continuity structure of the spectral features themselves. In addition, the models of traditional change detection also have great differences in performance, a poor generalization ability, low detection accuracy, obvious detection noise on different-quality sample sets, and other shortcomings, which mean they cannot meet the current demand for high-precision change detection [14].

Along with rapid advances in the technology for image processing and computer vision, research progress in machine learning has advanced the algorithms applied in the change detection domain. The object-oriented analysis thought in computer vision is applied to change detection. Ref. [15] proposed an unsupervised method, which directly calculates image differences in feature space, and used the kernel K-means algorithm and support vector machine to determine the change area. Ref. [16] studied the applicability of SVM in hyperspectral image change detection, proposed two combination frameworks of SVM, and verified the effectiveness of SVM in hyperspectral image change detection through the recognition rate and other indicators. In addition, support vector machines were designed for high-resolution images in [17], decision trees used for automatic recognition of change regions in [18], multi-core learning models designed in [19], and extreme learning machine used in [20]. These theories and methods have obtained a better accuracy in change detection. However, due to the large amount of computation and their complex forms, it is difficult to use these methods while simultaneously utilizing both image difference information and their own spatial information. Therefore, these methods do not have good generalization in different data distributions.

Given the advantages of deep learning in various fields and the advent of the remote-sensing big data era, deep learning has been introduced into remote-sensing image change detection. Ref. [21] overlaid two temporal images to generate fused features and implemented change detection by using a CNN. Ref. [22] used a CNN with a Siamese structure to extract features from images of two periods, respectively, and used matrix multiplication to realize feature fusion for feature maps extracted from two Siamese branches. In computer vision tasks, change detection is considered a semantic segmentation problem. In recent years, the change detection framework combining typical semantic segmentation models and Siamese networks has been widely applied in change detection tasks. Experiments have shown that the change detection model designed using this framework can achieve similar or even better qualitative and quantitative effects compared to existing specialized change detection models [23]. Ref. [24] proposed a processing method for a nested structure and new Siamese network features, improved the semantic segmentation network UNet++, and proposed a full convolutional Siamese network for change detection. Ref. [25] proposed the method of combining Siamese networks with an attention mechanism, and proposed a self-attention mechanism for change detection, which is used to model the spatiotemporal correlation between bi-temporal images and to update the feature map in the process of feature extraction to obtain features with time invariability. Ref. [26] used the high symmetry of hyperspectral images to propose a cross-time interactive symmetric attention network, extracted spatial–spectral features with an improved dual-attention mechanism, and obtained the time correlation of bi-temporal image pixels by using interactive symmetry of corresponding images. Existing change detection models based on deep Siamese networks use Siamese structures to extract features and have strong interpretability [25]. However, the utilization of spatial and spectral features is insufficient, and the spatiotemporal relationship between the changing data is ignored. Meanwhile, these methods perform poorly on datasets with extremely imbalanced samples.

Given the considerations above, this article proposes a hyperspectral image change detection model based on the balanced metric. Then, it establishes the model by combining the spatiotemporal attention mechanism and semantic feature extraction to construct the change detection method. The spatiotemporal attention mechanism is used to translate the bi-temporal hyperspectral images into the same feature space. Deep semantic features and shallow spatial features undergo semantic feature extraction. This method can effectively balance differential and self-spatial information, emphasizing the spatiotemporal relationship between changing data, thereby alleviating the problem of a poor performance in imbalanced sample datasets. The contributions of this article are summarized as follows:

(1) In response to the problem of joint representation of spatiotemporal spectral information in bi-temporal hyperspectral data, a spatiotemporal spectral attention mechanism is proposed, which focusing on spatiotemporal spectral features with time invariance.
(2) Addressing the problem of feature extraction and selection in hyperspectral data, a dual-branch feature extractor combining a semantic segmentation network and deep Siamese network is proposed, which simultaneously extracts spatial spectral joint features of bi-temporal hyperspectral data rich in semantic information.
(3) Considering the extremely uneven distribution of data types in hyperspectral image change detection tasks, metric learning is used to balance the contributions of different categories of data to the loss function.

The remainder of the article is structured as follows. In Section 2, the experiment’s datasets and methods are described. In Section 3, we give the experimental results. Section 4 presents a summary of our findings.

## 2. Materials and Methods

We use bi-temporal remote-sensing images $I_{1}$ and $I_{2}$ of size $H_{0}\times W_{0}$. The goal of change detection is to generate a label map named *M* that is equal in size to the input images. Every spatial position of *M* is assigned a changeable label. This paper focuses on binary change detection, so the label contains 1 (changed) and 0 (unchanged). However, some datasets contain unlabeled regions, and the change labels on these of the datasets are 2 (unlabeled areas), 1 (changed), and 0 (unchanged).

### 2.1. Overview

**Figure 1:**
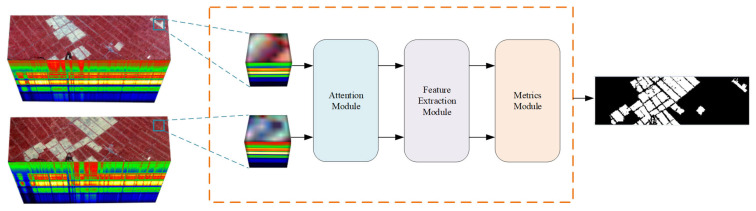
Flow block diagram.

Figure 1 shows the flow block diagram of this paper. The deep Siamese neural network consists of three modules: the attention module (Section 2.2), feature extractor (Section 2.3), and metrics module (Section 2.4). First, the original three-dimensional hyperspectral data are normalized and sliced, and the processed data are transformed into a hyperspectral dataset named $I_{0}=[I_{1},I_{2}]\in R^{C\times S\times S}$, which contains bi-temporal images called $I_{1}$ and $I_{2}$. This dataset has *N* samples of hyperspectral data blocks $N=H_{0}\times W_{0}$; each sample contains a pixel patch of size S×S in the region around the pixels, with C spectral segments. For each sample, in order to obtain attention feature pairs $A_{1},A_{2}\in R^{C\times S\times S}$, two hyperspectral data blocks are entered into the attention module simultaneously. Then, after the feature extraction module processes these feature maps, the feature vectors $X_{1},X_{2}\in R^{C_{1}}$ can be obtained, where $C_{1}$ is the channel dimension of the feature vectors. In the metrics module, we calculate the Euclidean distance between features for each sample pair in bi-temporal images $I_{1},I_{2}$, and a distance map *D* can be generated. In the training process, the model is optimized by minimizing the distance and batch-balanced contrastive loss of label maps, so that the large distance value is the changed point, and the small distance value is the unchanged point. In the process of testing, we use simple thresholding of the distance map to calculate the predicted label map *P*.

### 2.2. Attention Module

**Figure 2:**
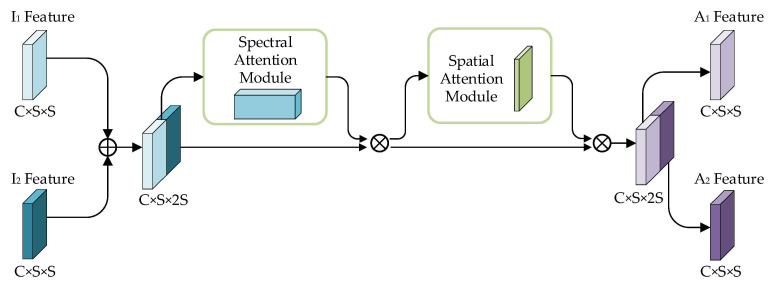
Attention module of the change detection model.

The inspiration for the attention mechanism comes from human research on biological systems [27]. It has a wide range of applications in various computer vision tasks, including object detection and image classification, and the effectiveness of the attention mechanism in modeling spatiotemporal relationships has been demonstrated [28]. The key concept of the attention mechanism is to determine the emphasis of images. Inspired by this, this paper applies the attention mechanism in hyperspectral image change detection. Through the attention module, the input tensors of the bi-temporal sample pair are translated into the same feature space. This attention mechanism eliminates the noise difference effect of the pictures taken at different times. It can capture abundant spatiotemporal relationships between sample pairs, which helps to obtain attention features rich in spatiotemporal–spectral information. The attention module is described in detail below:

The attention module used in this article is shown in Figure 2. The attention module stacks bi-temporal features $I_{1}$ and $I_{2}$ into a feature tensor $I\in R^{C\times S\times 2S}$. Then, the feature tensor is sequentially input into the channel attention module and the spatial attention module [29] to generate an updated feature tensor $A\in R^{C\times S\times 2S}$. Finally, it is split it into bi-temporal feature maps $A_{1},A_{2}\in R^{C\times S\times S}$.

In the channel attention module, global max pooling and global average pooling sequentially process the input feature maps, so that the input features $A\in R^{C\times S\times 2S}$ are compressed into channel descriptors of $A^{\prime }\in R^{C\times 1\times 1}$; in that way, the spatial information of bi-temporal feature map are compressed into the same spatial descriptor. Then, it passes the fully connected layer to produce an attention weight matrix. The features output by the fully connected layer are activated by a sigmoid to derive the update channel attention feature map. The input feature map is multiplied pixel by pixel with the channel attention feature map, and a calibrated attention feature map will be generated.

Equation (1) is the specific calculation process:

(1) $$M_{c}(F(X_{t}))=\sigma (MLP(AvgPool(F(X_{t})))+MLP(MaxPool(F(X_{t}))))X_{t}$$

where σ is the sigmoid operation, AvgPool is global average pooling, MaxPool is global max pooling, and MLP is the multilayer perceptron.

The input of the spatial attention module is the feature map, which is output from the channel attention module. First, global max pooling and global average pooling proceed, and these processed feature maps are connected to generate a valid feature descriptor $A^{\prime \prime }\in R^{S\times S\times 2}$, so that the bi-temporal input data are translated into the same feature space. Then, the dimension of the bi-temporal input data is decreased to a single channel, and these data are activated by the sigmoid to discern the spatial attention feature. At last, the spatial attention feature is multiplied pixel by pixel with the input feature of the module, and the final generated feature will be obtained and output.

Equation (2) is the specific calculation process:

(2) $$M_{s}(F)=\sigma (f^{7}([AvgPool(F);MaxPool(F)]))$$

where σ is the sigmoid operation, $f^{7}$ is a 2DCNN convolutional operation with a 7×7 convolutional kernel, AvgPool is global average pooling, and MaxPool is global max pooling.

### 2.3. Feature Extractor Module

Deep convolutional neural networks have been extensively used in remote-sensing tasks, including land-use classification, image super-resolution, and change detection [30]. The change detection framework combining typical semantic segmentation models and Siamese networks has been widely used in change detection tasks. Experiments have shown that the change detection model designed using this framework has similar or even better qualitative and quantitative effects than the existing change detection model [25]. The hyperspectral change detection task requires predicting images’ change labels pixel by pixel, which is a classification task. Therefore, this paper constructs a feature extractor using ResNet [31].

**Figure 3:**
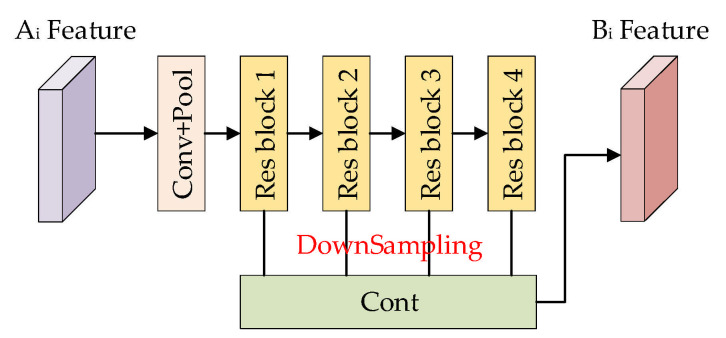
Feature extractor of the change detection model.

A feature extractor based on ResNet-50 [31] was designed in this study, as shown in Figure 3. The change detection task requires output change plots that are equal in size to the input image. For that reason, this paper omits the global pooling layer and the fully connected layer, used in primary ResNet to map image features to 1000-dimensional vectors. Deep-level feature semantics in convolutional neural networks are accurate but the spatial information is rough, while shallow-level feature spaces are rich in detail but lack semantic information. Therefore, deep-level semantic information will be combined with shallow-level spatial information to produce more detailed features in this paper. The feature extractor has four residual blocks. Each channel dimension is transformed into $C_{1}$. The output feature of each residual block is sent to different convolutional layers. Then, all feature maps are resized to size 1×1. In this way, four groups of feature maps can be obtained from various phases of this network. We concatenate these four groups of feature maps in the channel dimension (the result is $1\times 1\times 4C_{1}$) and send the result to the convolutional layer to produce the final feature vector ($1\times 1\times C_{2}$). This convolutional layer can generate more discriminative and representative features by reducing the feature channel dimension. In the experiment, after a trade-off, $C_{1}$ and $C_{2}$ are separately set to 96 and 64 to give consideration to efficiency and accuracy, respectively.

### 2.4. Metrics Module

#### 2.4.1. Deep Metrics Learning

Deep metrics learning trains the network to study nonlinear transformations from input to embedded space, which means the feature tensor of homologous samples is close while the feature tensor of different samples is far apart. Change detection methods based on deep metrics learning have achieved a good performance over the last couple of years. This paper optimizes the model by minimizing distance and label loss, improves comparison loss, and makes the distance of inter-class features long, while the distance of intra-class features is short. In each iteration of the training phase, given a sample feature pair $X_{1},X_{2}\in R^{C_{1}}$, we calculate the Euclidean distance of feature vectors in the embedding space. We learn the network parameters by improving contrastive loss so that the distance between changed pixels is large while the distance between unchanged pixels is small. In the process of testing, we predict change labels through fixed threshold segmentation.

Equation (3) is the Euclidean distance of tensors $F_{1}$ and $F_{2}$:

(3) $$dist={\left\|F_{2}-F_{1}\right\|}_{2}$$

where $F_{1}$ is the hyperspectral feature vector of the first temporal image, and $F_{2}$ is the hyperspectral feature vector of the second temporal image. The automatic learning process of the distance is achieved by training two branches of a convolutional neural network that share the same weights. If the two hyperspectral data blocks are similar, the distance between the two tensors is short; otherwise, if they change, the distance is long.

In the process of testing, we derived the change map *P* through fixed threshold segmentation.

Equation (3) is the threshold division process:

(4) $$P_{i,j}=\left\{\begin{array}{l} 1\;D_{i,j}\geq \theta \\ 0\;else \end{array}\right.$$

*i* and *j* are the indexes of the pixel abscissa and pixel ordinate, respectively. The threshold θ is set to 1. The pixels marked as 1 are recognized as change pixels in the changed map *P*, and the pixels marked as 0 are recognized as unchanged pixels.

#### 2.4.2. Loss Function

Most multi-classification tasks in machine learning generally have imbalance issues [32]. For change detection in remote-sensing images, there is a significant difference in the number of changed and unchanged pixels. Usually, the changed pixels only account for a small portion of all pixels, and this can cause bias in the network in the process of training. This article cites the batch-balanced contrastive loss (BCL) [25] to reduce the impact of class imbalances and uses batch weights instead of the original class weights of the contrastive loss. Given a bi-temporal sample pair ($I_{1},I_{2},M$), $I_{1},I_{2}\in R^{C\times S\times S}$ and M∈R, where $I_{1},I_{2}$ are bi-temporal samples and *M* is a label map. The sample distance D∈R is acquired by means of the attention module and the feature extraction module.

Equation (4) is the definition of BCL:

(5) $$L(D,M)=\frac{1}{n_{u}}\sum_{b,i,j}\left(1-M_{b,i,j}^{*}){D_{b,i,j}^{*}}^{2}+\frac{1}{n_{c}}\sum_{b,i,j}M_{b,i,j}^{*}Max(0,m-{D_{b,i,j}^{*}}^{2}\right)$$

where *D* expresses the distance map composed of the Euclidean distance of a training batch of bi-temporal hyperspectral images. *M* represents the label map composed of the category labels of a training batch of bi-temporal hyperspectral images. $D_{b,i,j}^{*}$ represents the elements in distance map *D*. $M_{b,i,j}^{*}$ expresses the elements in the label map *M*. *b* represents the training batch. *I* and *j* stand for the indexes of the pixel abscissa and pixel ordinate, respectively. $n_{u}$ and $n_{c}$ represent the number of changed and unchanged pixels, respectively. *m* represents the threshold. The following presents the entire algorithmic process of the model (Algorithm 1).
**Algorithm 1.** The following presents the entire algorithmic process of the model
Input: The raw two phase hyperspectral data X_1_, X_2_ and ground truth X_R_ Output: Change detection results of each pixel is compared with the overall change detection map. 1: Trim the bi-temporal hyperspectral datasets and divide them into training sample pairs and testing sample pairs. 2: Splicing the dual temporal hyperspectral images along the spatial direction and passing them through a spatiotemporal attention module to focus on spatial spectral features with temporal invariance. 3: A dual branch feature extractor that combines semantic segmentation network and deep twin network to extract features from two time periods, emphasizing rich semantic information. 4: Calculate the Euclidean distance between two temporal features with rich semantic information. 5: Calculate the balance metric loss function based on Euclidean distance and perform binary classification of features. 6: Use the trained model to perform change detection on the test set and generate a change detection result map.

### 2.5. Dataset Description

**Figure 4:**
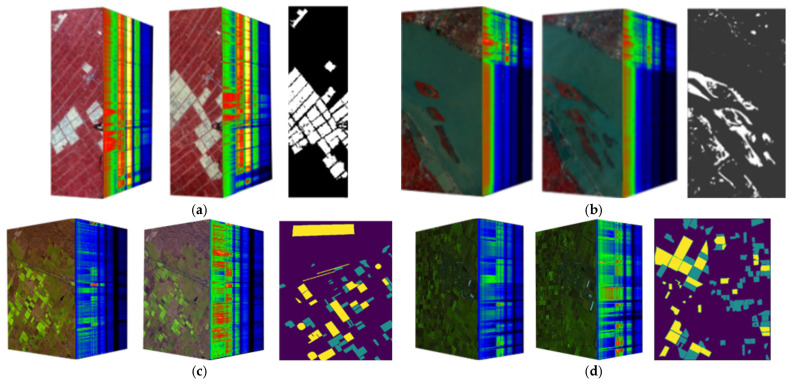
The datasets used in this paper: (**a**) Farm dataset; (**b**) River dataset; (**c**) Babara dataset; (**d**) Bayarea dataset.

The first dataset used in the experiment, Farm, includes two hyperspectral images for change detection, which were, respectively, collected on 3 May 2006, and 23 April 2007. The images offer data of wet agricultural areas of Yancheng, Jiangsu Province, acquired by the Earth Observing-1 (EO-1) sensor with 242 spectral bands. The spatial resolution of the sensor is about 30 m, covering a spectrum with a wavelength of 0.4–2.5 microns, with a spectral resolution of 10 nanometers. Due to interference from atmospheric water vapor, the collected data exhibit a low signal-to-noise ratio. After removing the bands affected by water vapor interference, the experiment used the dataset size of 450×140 pixels and 155 bands. Figure 4a shows the dataset and ground-truth images.

The second dataset used in the experiment, River, includes two hyperspectral images for change detection, which were collected on 3 May 2013 and 31 November 2013, respectively, offering data on changes in the river basin of a place in Jiangsu Province. The images were collected by the EO-1 sensor. Based on existing research and ENVI analysis, spectral segments with a high signal-to-noise ratio were selected for experimentation in this study. The experiment used the dataset size of 463×241 pixels and 198 bands. Figure 4b illustrates the dataset and ground-truth images.

The third dataset, Babara, includes two hyperspectral images for change detection, which were, respectively, collected in 2013 and 2014 in Santa Barbara, California. The images were collected by an Airborne Visible Infrared Imaging Spectrometer (AVIRIS) sensor with 224 bands. The AVIRIS data provide a spatial resolution of 20 m, covering a spectrum with a wavelength of 0.4–2.5 microns, with a spectral resolution of 10 nanometers. The experiment used the dataset size of 987×740 pixels and 224 bands. Figure 4c shows dataset images and ground-truth images. In the figure, purple indicates unlabeled pixels, cyan indicates unchanged pixels, and yellow indicates changed pixels.

The fourth dataset used in the experiment, Bayarea, includes two hyperspectral images for change detection, which were, respectively, collected in 2013 and 2015 in Patterson, California. The images were collected by the AVIRIS sensor. The experiment used the dataset size of 600×500 pixels and 224 bands. Figure 4d shows the dataset and ground-truth images. In the figure, purple indicates unlabeled pixels, cyan indicates unchanged pixels, and yellow indicates changed pixels.

### 2.6. Implementation Details

#### 2.6.1. Criteria

This paper will use the confusion matrix to obtain the classification accuracy, precision, recall, missing alarm, and false alarm as evaluation criteria. See Table 1.

Classification accuracy = $\frac{X_{11}+X_{22}}{N}$, which reflects the proportion of samples judged to be correct in all samples.

Precision = $\frac{X_{11}}{X_{1+}}$, which reflects the proportion of true positives in the positive samples.

Recall = $\frac{X_{11}}{X_{+1}}$, which reflects the ratio of correctly judged positive samples in all positive samples.

Missing alarm = $\frac{X_{21}}{X_{+1}}$, which reflects how many positive samples have been missed.

False alarm = $\frac{X_{12}}{X_{+1}}$, which reflects how many of the samples judged as positive were wrong.

F1-score = $\frac{2\times Precision\times Recall}{Precision+Recall}$, which is the harmonic mean of precision and recall, an important evaluation metric for binary classification tasks.

#### 2.6.2. Dataset

This paper splits the dataset into two parts at random, selecting 10% of each class of samples for training and the remaining 90% for testing. Each sample pixel block in the dataset contains neighborhood pixels in a range of 7×7 around it.

#### 2.6.3. Training Setting

The implementation of this method is based on Pytorch 1.10.0 [33]. This model was fine-tuned based on the ResNet-50 [31] model, which was pre-trained by ImageNet. The initial learning rate is $1\times 10^{-3}$. In the first 30 epochs, we maintained the same learning rate, while in the remaining 70 epochs, we gradually decreased it linearly to 0. This study adopted the Adam optimizer [34], where the batch size was 32.

#### 2.6.4. Comparison Method

CNN: The input of this network is a difference map between bi-temporal sample pairs. The network structure is the same as the feature extractor based on Resnet-50, while the residual structure is deleted, and the 2D convolutional layer is followed by the feature extractor to reduce the changing feature to 1 dimension. In the process of training, the network parameters are learned using contrast loss so that the distance between changed pixels is large while the distance between unchanged pixels is small. In the process of testing, the predicted change labels are obtained by means of a fixed threshold segmentation.

Siam-Resnet: The input of this network comprises bi-temporal sample pairs. The network has two branch structures, each the same as for the Section 2.3 feature extractor, and the feature extractor is followed by the metrics module to output the Euclidean distance of their features. During the training stage, network parameters are studied through contrastive loss, so that the distance between changed pixels is large while the distance between unchanged pixels is small. During the testing stage, the predicted changed label is obtained by means of a fixed threshold segmentation.

## 3. Results and Discussion

This section thoroughly evaluates the model presented in this paper and makes a comparison with several other change detection methods. Our experiments were conducted on the Farm, River, Babara, and Bayarea datasets.

### 3.1. Experiment on Farm Dataset

Attention Module Ablation Experiment: Verify the effectiveness of the attention module by comparing Siam-Resnet with the method presented in this article. The ablation experiment of the Farm testing dataset is shown in Table 2. The performance of our method can be evaluated by calculating the classification accuracy, precision, recall, and F1-score. We can observe that the method in this paper of adding an attention module is significantly improved compared to Siam-Resnet in the classification accuracy, precision, and recall. Meanwhile, the attention module has a better effect when using a convolutional kernel of size 7×7 than when using a convolutional kernel of size 3×3 on the Farm dataset.

Batch-balanced contrastive loss ablation experiment: The BCL used in this paper is helpful to alleviate imbalances of class. Table 3 shows the BCL ablation experiment on the Farm testing dataset. It can be observed that compared with traditional contrastive loss, the performance of various models (CNN, Siam-Resnet, method in this paper) is improved with BCL. In each training iteration, the contribution of minority (changed) samples and majority (unchanged) samples to loss is dynamically balanced with BCL. It can reduce the likelihood that the network will skew toward a certain category, thus solving the problem of imbalanced sample data in different categories in current hyperspectral image change detection and bringing an improvement in model performance.

**Figure 5:**
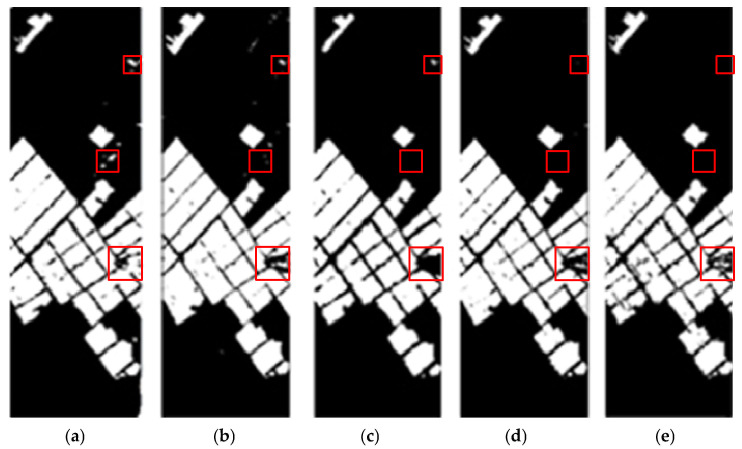
Comparison of change detection effects on Farm dataset, the red box highlights the differences in the detection results. (**a**) Change detection result of CNN model; (**b**) change detection result of Siam-Resnet model; (**c**) change detection result of CSA-net model; (**d**) change detection result of the method in this paper; (**e**) ground-truth map.

Figure 5 illustrates the comparison of various change detection methods. The triangle part on the right edge of the CNN prediction map is significantly different from the label map. At the same time, there is a large amount of white noise in the prediction map, and there is a serious false alarm phenomenon. The prediction map generated by Siam-Resnet has many continuous banded noise bars, and the noise bar occurrence area is similar to the noise region of the CNN prediction map. This is because neither the CNN method nor the Siam-Resnet method can eliminate the misjudgment of the same region caused by the noise difference in images taken at different times. The edge of the prediction map produced by the CSA-Net method is clear and continuous. The model learns the joint features of the time–space spectrum through the cross-time interactive attention module, but it is easy to dilute the differences between different samples in the process of complementing the features in the time domain. This can result in discriminating the changed pixels of the entire region as unchanged pixels, such as the triangle part of the right edge of the prediction map. It can be observed that more detailed results are obtained with this article’s method. The spatiotemporal attention module is used to learn local spatiotemporal relationships between the corresponding pixel blocks, and these relationships are used to obtain better features.

**Figure 6:**
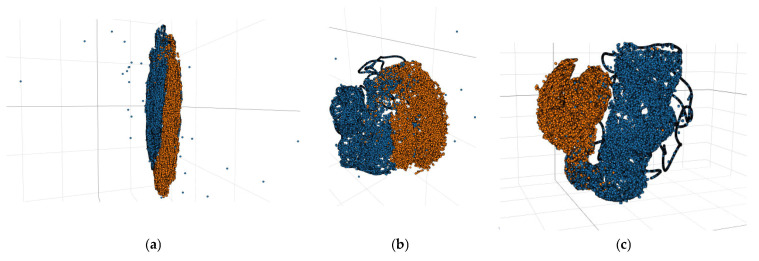
Feature visualization comparison diagram of the three methods. (**a**) t-SNE feature extracted by CNN method; (**b**) t-SNE feature extracted by Siam-Resnet method; (**c**) t-SNE feature extracted by the method in this paper.

The deep features extracted by CNN, Siam-Resnet, and the method in this paper, respectively, on the Farm dataset were processed via dimensionality reduction and visualization by the method of t-SNE. Figure 6 shows the comparison result. Compared with the CNN method and Siam-Resnet method, the method in this paper has a larger distance between changed and unchanged pixels and extracts more representative features. It can be observed that the spacing of the feature classes extracted by CNN and Siam-Resnet is small, and there is partial aliasing. In this paper, the extracted feature classes’ spacing is greater and outliers are fewer, and the method can separate changed and unchanged pixels more effectively.

**Figure 7:**
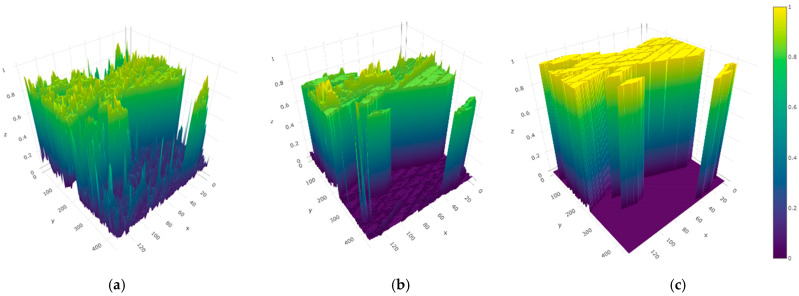
Measurement feature comparison diagram of the two methods. (**a**) Measurement feature of Siam-Resnet method; (**b**) measurement feature of the method in this paper; (**c**) measurement feature of the label map.

To analyze the feature extraction performance levels of various methods in the class imbalance dataset more intuitively, we use the Farm dataset as an example for visual analysis. Figure 7 shows the metric feature visualization comparison results of Siam-Resnet and this article’s method. Compared with Siam-Resnet, the features extracted by the method in this paper are more differentiated and more conducive to the threshold division of the measurement module. The metric characteristics extracted by Siam-Resnet, shown in the figure, have many noise peaks. This is not conducive to the threshold division of the measurement module. The metric characteristics extracted in this methodology are closer to the visualization results of the real label map, so the network in this paper can achieve a better performance.

### 3.2. Experiment on River, Babara, and Bayarea Datasets

**Figure 8:**
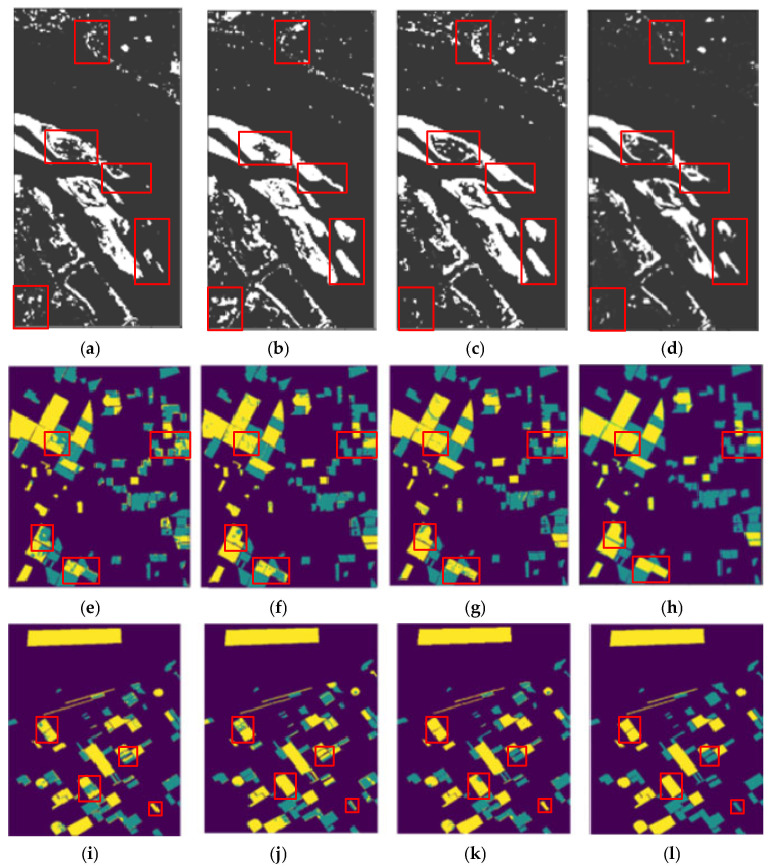
Change detection results on different datasets. (**a**) CNN result on River dataset; (**b**) Siam-Resnet result on River dataset; (**c**) this paper’s method’s result on River dataset; (**d**) ground truth of River dataset; (**e**) CNN result on Bayarea dataset; (**f**) Siam-Resnet result on Bayarea dataset; (**g**) this paper’s method’s result on Bayarea dataset; (**h**) ground truth of Bayarea dataset; (**i**) CNN result on Babara dataset; (**j**) Siam-Resnet result on Babara dataset; (**k**) this paper’s method’s result on Babara dataset; (**l**) ground truth of Babara dataset.

The performance of the method in this article is also evaluated on several other datasets and compared to CNN and Siam-Resnet. Figure 8 illustrates the performance of change detection by various methods on different datasets. This paper’s model can obtain more accurate and smoother results than other methods. Figure 8 illustrates the performance data comparison of various methods on three datasets. It can be observed that the method proposed in this paper consistently outperforms other methods in precision, missing alarm, and false alarm.

Figure 8 illustrates the result on three datasets, where this article’s method consistently performs more accurately and smoothly and the false alarm is lower than for other methods. On the River dataset, as shown in Figure 8a,d, for some unchanged regions, CNN and Siam-Resnet incorrectly detect them as changed regions, such as the circular beach in the lower left part and in the middle of the figure. This paper’s method can learn the data distribution of hyperspectral images, eliminate the influence of noise, and obtain more effective feature representation, so as to achieve a better change detection effect. The Bayarea dataset is a change detection dataset of urban scenes. As shown in Figure 8e, CNN method detects some unchanged regions as changed regions, for example, the part of the figure on the right side where the change labels are scattered. Figure 8f shows that the Siam-Resnet method has a false check of the entire block in the lower edge of the figure. Figure 8g shows that this method is more sensitive to contour changes, and the distinction between the intersection of the changed area and the unchanged region is smoother and more accurate. On the Babara dataset, as shown in Figure 8i, the CNN method has a large number of false detection areas. Figure 8j shows that Siam-Resnet contains many cyan noise spots. Figure 8h shows that the method in this paper eliminates the atmospheric influence and environmental noise of taking hyperspectral images at different times, because it learns the space–time-spectral joint information of hyperspectral images and obtains a more effective feature representation and better prediction maps.

**Figure 9:**
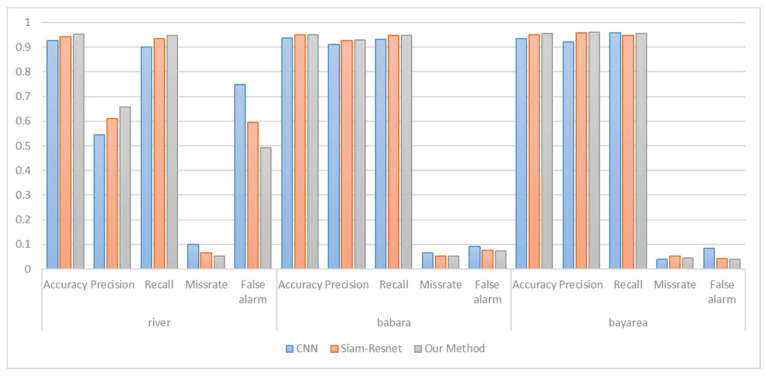
Change detection performance comparison on three datasets.

Figure 9 illustrates the performance comparison of various methods on three datasets. The change detection task requires both high precision and a low missing alarm. The method proposed in this paper is always better than other methods in terms of precision, missing alarm, and false alarm. It can accomplish the task of change detection better. Compared with Siam-Resnet, the classification accuracy was improved by an average of 0.83%, and the missing alarm was reduced by an average of 0.77% on the three datasets. Compared with CNN, the classification accuracy was improved by an average of 2.16%, and the missing alarm was reduced by 2.05% on average.

## 4. Conclusions

This paper takes hyperspectral images as the research object and proposes a hyperspectral image change detection model based on a deep Siamese network. Bi-temporal hyperspectral images are translated into the same feature space using an attention module, excluding the effects of atmospheric differences and noise in bi-temporal conditions. Deep semantic features and shallow spatial features are extracted using the semantic segmentation network Resnet. We measure sample features using the Euclidean distance. In the process of training, we optimize the model by minimizing distance and label loss. In the process of testing, labels are predicted by simple thresholding of the distance map.

The experiments were performed on four datasets: Farm, River, Bayarea, and Babara. Compared with the methods based on CNN and Siam-Resnet, the method in this paper obtained a higher classification accuracy, lower missing alarm, and lower false alarm. The prediction maps generated by this method were more detailed and smoother, with less noise. Ablation experiments were conducted on the attention module and BCL module of the Farm dataset. The experiments showed that the attention module significantly improved model performance. In the attention module, compared with the convolutional kernel of size 3×3, the convolutional kernel of size 7×7 had better results. In CNN, Siam-Resnet, and the method of this paper, the BCL module can obtain good change detection indicators and improve the performance of the model.

Future and Prospects: Hyperspectral data produce difficulties in obtaining labeled samples. In this experiment, 10% of the samples were used for training, and 90% of samples for testing. However, there is still a problem associated with the high cost of sample labeling in practical applications. The next target is to build a suitable unsupervised hyperspectral image change detection method and find a suitable abnormal sample removal method to increase the accuracy and stability of its prediction.

## Figures and Tables

**Table 1:** Confusion matrix.

	Real Images			
**Predicted Images**	**Pixel type**	**Changed**	**Unchanged**	**Row Total**
	**Changed**	$X_{11}$	$X_{12}$	$X_{1+}$
	**Unchanged**	$X_{21}$	$X_{22}$	$X_{2+}$
	**Column Total**	$X_{+1}$	$X_{+2}$	*N*

**Table 2:** Ablation experiment of attention module.

Module	Kernel Size	Classification Accuracy (%)	Precision (%)	Recall (%)	F1-Score (%)
**Siam-Resnet**		96.05	95.87	92.82	94.32
**Method in this paper**	3×3	97.31	96.29	94.54	95.41
**Method in this paper**	7×7	97.45	96.48	94.84	95.63

**Table 3:** Ablation experiment of BCL module.

Module	Loss Function	Classification Accuracy (%)	Precision (%)	Recall (%)	F1-Score (%)
**CNN**	Contrastive loss	94.14	93.29	92.36	92.82
**CNN**	BCL	94. 58	93.43	92.55	92.99
**Siam-Resnet**	Contrastive loss	95.71	94.90	92.26	93.56
**Siam-Resnet**	BCL	96.05	95.87	92.82	94.32
**Method in this paper**	Contrastive loss	97. 23	96.33	94.59	95.45
**Method in this paper**	BCL	97.45	96.48	94.84	95.65

## Data Availability

Data are contained within this article.
